# The Derived Allele of *ASPM* Is Associated with Lexical Tone Perception

**DOI:** 10.1371/journal.pone.0034243

**Published:** 2012-04-17

**Authors:** Patrick C. M. Wong, Bharath Chandrasekaran, Jing Zheng

**Affiliations:** 1 Roxelyn and Richard Pepper Department of Communication Sciences & Disorders, Northwestern University, Evanston, Illinois, United States of America; 2 Department of Otolaryngology, Head and Neck Surgery, Northwestern University, Chicago, Illinois, United States of America; 3 Hugh Knowles Center for Clinical and Basic Science in Hearing and Its Disorders, Northwestern University, Evanston, Illinois, United States of America; Wake Forest School of Medicine, United States of America

## Abstract

The *ASPM* and *MCPH1* genes have been implicated in the adaptive evolution of the human brain [Mekel-Bobrov N. et al., 2005. Ongoing adaptive evolution of ASPM, a brain size determinant in homo sapiens. *Science* 309; Evans P.D. et al., 2005. Microcephalin, a gene regulating brain size, continues to evolve adaptively in humans. *Science* 309]. Curiously, experimental attempts have failed to connect the implicated SNPs in these genes with higher-level brain functions. These results stand in contrast with a population-level study linking the population frequency of their alleles with the tendency to use lexical tones in a language [Dediu D., Ladd D.R., 2007. Linguistic tone is related to the population frequency of the adaptive haplogroups of two brain size genes, ASPM and microcephalin. *Proc. Natl. Acad. Sci. U.S.A*. 104]. In the present study, we found a significant correlation between the load of the derived alleles of *ASPM* and tone perception in a group of European Americans who did not speak a tone language. Moreover, preliminary results showed a significant correlation between *ASPM* load and hemodynamic responses to lexical tones in the auditory cortex, and such correlation remained after phonemic awareness, auditory working memory, and non-verbal IQ were controlled. As in previous studies, no significant correlation between *ASPM* and cognitive measures were found. *MCPH1* did not correlate with any measures. These results suggest that the association between the recently derived allele of *ASPM* is likely to be specific and is tied to higher level brain functions in the temporal cortex related to human communication.

## Introduction

The *ASPM* and *MCPH1* genes are expressed in cerebral cortical ventricular and proliferative zones [1] and play crucial roles for normal neurogenesis [2]–[4]. Although they have been implicated in the adaptive evolution of the human brain [5], [6], leading to the hypothesis that the derived alleles of these two genes contribute to human cognition, empirical support for such a hypothesis remains sparse. Experimental findings have yet to link *ASPM* A44871G (rs41310927,Ser2562Gly) and *MCPH1* G37995C (rs930557, Asp314His) with broad measures of human cognition phenotypes, including working memory [7] and broad mental abilities [8]. Furthermore, no connection has been found between these alleles and head circumference [9] or brain size in either adults or children [7], [10]. Although more recent studies have found sex-dependent association between brain structures and *ASPM* and *MCPH1* [11]–[12], the SNPs in question were not the ones implicated in the adaptive evolution [5]–[6]. One interpretation of these null findings is that these genes are not related to adaptive evolution of human brain function. Alternatively, these genes could be related to more specific neural phenotypes (e.g., spoken language processing) and endophenotypes (e.g., neurophysiology of the auditory temporal cortex) that have yet to be examined.

The evolutionary increase in brain size and the emergence of language capacity in humans have been subjects of inquiry [13]–[15]. As the vast majority of the world’s languages are spoken languages, it is especially important to examine brain functions that are specific to spoken language such as speech sound perception. Lexical tones are pitch patterns that are used to mark word meaning and can be found in most spoken languages of the world according to some estimates [16]; these include at least half of the world’s languages [17]. For example, in Mandarin Chinese, a tone language, level, rising, and falling pitch patterns superimposed on the syllable/ma/mean ‘mother,’ ‘hemp,’ and ‘to scold,’ respectively, whereas such word-level tonal contrast is absent in non-tone languages such as English.

A clue to a possible connection between *ASPM*/*MCPH1* and speech sound perception is a population study by Dediu and Ladd [18] in which a significant correlation was found between the frequency of derived alleles and a tendency towards use of lexical tones in a language, even after geographical and historical factors were controlled. An explanation for the Dediu and Ladd findings [18] is that *ASPM* and *MCPH1* are associated with the processing of simultaneous acoustic cues within a short temporal window, as lexical tone speakers are required to process both the syllable and pitch as an integral unit. An examination of the association between these genes and lexical tone processing, a specific linguistic function, would mitigate the potential difficulties faced by earlier studies of broad phenotypic cognitive measures.

The Dediu and Ladd population findings provide the basis of our experimental study: there is an association between *ASPM*/*MCPH1* genes and lexical tone perception. In the present study, we tested native speakers of American English of European descent (Caucasians) who had not been exposed to a tone language. Non-tone language speakers were chosen because they allowed for the examination of a genotype-phenotype association without the influence of significant environment (linguistic) factors. Furthermore, non-tone language speakers show highly variable responses in tone perception, which has been attributed to neurophysiological and neuroanatomical factors [19], [20]. Subjects were tested on a Tone Perception test [21] and a subgroup of these subjects who were recruited at a separate time also participated in an fMRI adaptation (fMRI-A) experiment in which they processed lexical tones. As will be discussed in the Results section, we analyzed our behavioral results by considering gene-behavioral correlations with the two subgroups of subjects combined, as well as separately, in an attempt to replicate our behavioral findings. The Tone Perception test consisted of identifying resynthesized level, rising, and falling tones (resembling Mandarin Chinese) embedded in vowels produced by multiple talkers. In the fMRI experiment, participants performed an orthogonal loudness judgment task while listening to lexical tones presented in two conditions. In the repeat condition, tones were presented repetitively within a block. In the tone-change condition, different tones were presented within a block (see Methods for details). Previous studies have shown that repetitive presentation of stimuli reduces the BOLD response in regions sensitive to the feature being repeated, a phenomenon referred to as repetition suppression [22], [23]. When features of the stimuli are changed, no such response reduction is found. Response suppression to repeating features is argued to indicate increased neuronal efficiency in the processing of particular features (those that are repeated). From a mechanistic perspective, current models predict that with every repetition, neuronal encoding becomes sharper (sharpening hypothesis), i.e., fewer neurons encode the relevant feature, or neural responses become faster (facilitation hypothesis), both of which may lead to a reduction in BOLD response [23], [24]. fMRI-A designs offer an objective way of examining neural representation of stimulus features of interest without the confound of overt processing. In our case, the feature of interest was lexical tone but the subjects were asked to perform a loudness judgment task that was orthogonal to the processing of lexical tones. We expected a significant negative relationship between BOLD activity in the repeat condition and *ASPM,* and/or *MPCH1* gene load, the number of derived alleles. Such results would indicate that individuals who have a higher gene load would demonstrate more efficient (i.e., more response suppression in the repeat condition) neural encoding of tones.

## Methods

This study is approved by the Northwestern University Institutional Review Board. All participants provided informed written consent in accordance with the Institutional Review Board, Northwestern University. This study examines a gene that has been previously identified and no new genetic information has been generated.

### Participants

Thirty-two younger adults (less than 35 years old) who reported to be Caucasians participated in this study. All subjects were native speakers of American English with no previous experience with any tone languages. All subjects passed a hearing screening at 30 decibel Hearing Level for the frequencies of 500, 1000, 2000, and 4000 Hz. All participants were tested on the Tone Perception test, as well as Sound Blending (SB) and Auditory Working Memory (AWM) tests of the Woodcock Johnson-III Tests of Cognitive Abilities [25] for assessing phonemic awareness and working memory, respectively. As a preliminary study examining the neural basis of *ASPM*, a subgroup consisting of 13 subjects (7 females) between 21 to 34 years old (mean  =  25.54) also participated in an fMRI adaptation (fMRI-A) experiment in which they processed lexical tones [Non-fMRI subjects consisted of 19 subjects (16 females) between 18 to 27 years old (mean  =  20.5)]. Subjects who participated in the fMRI-A experiment were also tested on the Test of Nonverbal Intelligence (Third Edition) for nonverbal IQ [26]. While non-fMRI subjects participated in various other shorter experiments in addition to the current study, the fMRI subjects all agreed to and completed participation in a separate language training experiment involving 9 days of training, 10 days of imaging (ERP and MRI), and 2 hours of cognitive and language testing over a two-week period. Note that the Tone Perception and fMRI data reported here were collected before the other experiments.

### Tone Perception

The Tone Perception test is identical to the one used in previous studies [21], [27]. Subjects heard resynthesized versions of the Mandarin vowels/a/,/i/,/o/,/e/, and/y/with Mandarin Tones 1 (level), 2 (rising), and 4 (falling) superimposed. These vowels were produced by two male and two female native Mandarin speakers. For each stimulus, subjects were asked to indicate its pitch pattern by selecting the appropriate arrows depicted on a computer screen (i.e., →  =  level, ↑  =  rising, and ↓  =  falling). For each trial, one target and one distractor picture was shown. For example, if the trial is an/a/spoken with the level tone, subjects saw → (left) and ↑ (right) on the screen. Subjects used a button box to indicate their responses (the correct response in the case would be the arrow displayed on the left). Subjects performed this test in a sound-attenuated chamber.

### fMRI Procedures

Participants performed an orthogonal loudness judgment task while listening to the four Mandarin tone categories (Tone 1 (T1), Tone 2 (T2), Tone 3 (T3), and Tone 4 (T4) that are phonetically described in terms of pitch as “high-level”, low-rising, low-dipping, and high-falling respectively) in the MRI scanner. This orthogonal task was used to ensure that participants were attentive throughout the experiment. Loudness judgment does not interfere with pitch processing, as seen in previous studies [28], [29].

In the fMRI experiment, lexical tones were presented in two conditions. In the repeat condition, the speech sound containing a tone was repeated four times (e.g. T1, T1, T1, T1), within a TR (repetition time) of 12 seconds. Therefore, in the repeat condition, the same pitch pattern (e.g. high-level, high-level, high-level, high-level) was repeated. All four tone categories were used with equal probability. In the tone-change condition, the tone varied within the TR of 12 s (e.g. T4, T1, T2, T3). Therefore, in this condition, the pitch pattern varied within a TR (e.g. high-falling, high-level, low-rising, low-dipping). The order of the tones was randomized within each TR. Irrespective of condition, the task was to indicate if the second stimulus was louder or softer than the first stimulus. Regression analysis was conducted separately for the two conditions. As a control, we expected no relationship between BOLD activity and *ASPM* allele load for the tone-change condition (since tone information is constantly changing, no repetition suppression to tones was expected). Since participants performed at ceiling during the loudness judgment task for both conditions, task performance was not considered as a factor during analyses.

**Figure 1:**
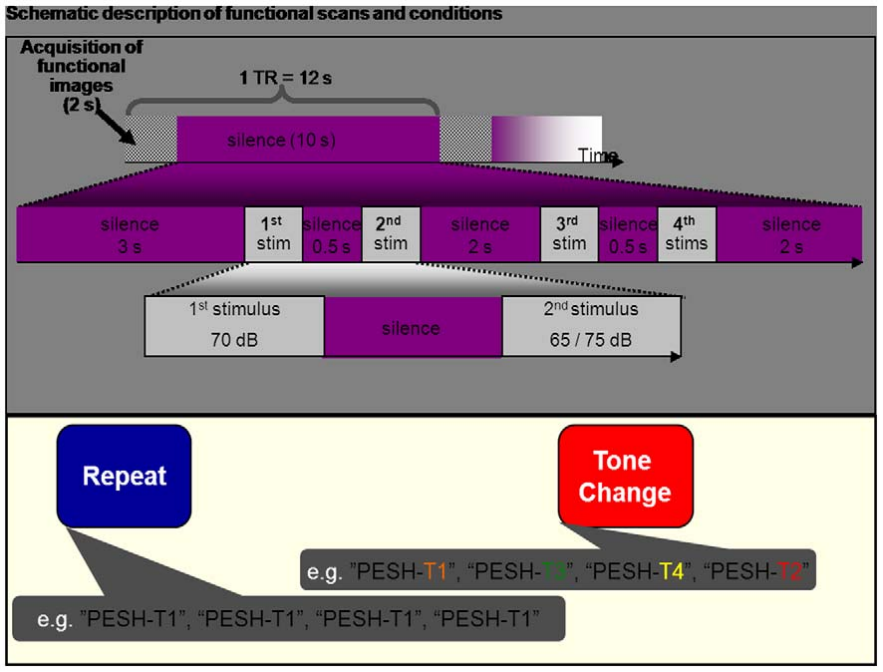
Schematic description of functional scans and conditions. Top panel shows the sparse sampling design used during functional scans. Participants heard four sounds presented within a 10-sec noise-free window. Functional images were acquired during the first two seconds. Participants were asked to judge whether the 2^nd^ and 4^th^ stimuli were louder/softer than the 1^st^/3^rd^. The first and third stimuli were always presented at fixed intensity level. The 2^nd^ and 4^th^ stimuli were either 5 dB louder or 5 dB softer. In repeat conditions, participants heard four repetitions of the same pseudoword that contained the same pitch pattern. In tone change conditions, the same pseudoword was presented, but with different pitch patterns.

Magnetic Resonance Images were acquired using a Siemens 3T Trio MRI scanner. For each participant, a high resolution, anatomical T1-weighted 3D volume was acquired axially (MP-RAGE; TR  =  2300 ms; Echo Time (TE)  =  3.36 ms, flip angle  =  9°, TI  =  900 ms, matrix size  =  256×256, FOV of 22 cm, slice thickness  =  1 mm). Functional T2*-weighted images were acquired axially using a susceptibility weighted EPI pulse sequence (TE  =  20 ms, TR  =  12 s, flip angle  =  90°, in-plane resolution  =  3.4375 mm×3.4375 mm, 38 slices with a slice thickness  =  3 mm (without gap between slices) were acquired in an interleaved measurement). A sparse sampling method was used, which allows for stimuli presentation in silence [19], [28] (see Figure 1). This design allowed participants to hear the stimuli without the interference of scanner noise and provides adequate time for the scanner-noise induced hemodynamic response (HR) to reduce and not overlap with the HR to the auditory stimuli. There were 40 blocks per condition and 40 blocks of silent trials (randomized). The null trials were used to establish baseline. The functional MR images were analyzed using AFNI [30] images and were corrected for motion and slice-time, and spatial smoothing (FWHM 6 mm) was performed, followed by linear detrending and resampling to a resolution of 3 mm^3^. Square waves modeling the events of interest were created as extrinsic model waveforms of the task-related hemodynamic response. The waveforms of the modeled events were then used as regressors in a multiple linear regression of the voxel-based time series. Normalized beta values signifying the fit of the regressors to the functional scanning series, voxel-by-voxel for each condition, were used for group analyses. Anatomical and functional images from each subject were normalized to a standard stereotaxic template (ICBM 152). To examine the effect of allele load on BOLD responses to tones, at the second (group) level, we conducted multiple regression analysis performed using the program 3DRegAna (http://afni.nimh.nih.gov/pub/dist/doc/program_help/3dRegAna.html) implemented within AFNI [30] on brain activation in the repeat > silence and change > silence conditions. Two separate regression analyses, one using covariates (IQ, sound blending, auditory working memory scores) and one without covariates. We used *ASPM* load, *MCPH1* load, and *MCPH1/ASPM* load as regressors. For these various analyses, individual voxel thresholds were set at a p < 0.05 (corrected for multiple comparisons). Corrected threshold was determined based on a Monte Carlo simulation (using AFNI function AlphaSim) of significant voxels within the contrast “all listening conditions” minus “silent conditions”. The deactivation network was excluded to avoid artifacts generated from significant deactivation during the listening task, consistent with the previous studies that examined repetition suppression of auditory word information [28], [31]. Table 1 shows regions that were significantly active (p < 0.05, corrected) obtained from the regression analyses.

**Table 1:** Association between load of *ASPM-G* allele and neural repetition suppression to tone repeat (1) and tone-change (2) conditions.

	Activation peak	Talairachcoordinates x,y,z	Cluster size (voxels)	Peak T value	Peak *r* value (p-value)
***(1a) Repeat condition***	Right transverse temporal gyrus/superior temporal gyrus	39, −33, 13	370	−5.9	−0.89 (.000046)
***(1b) Repeat condition (with*** ***covariates*** * ***)***	Right transverse temporal gyrus/superior temporal gyrus	39, −34, 17	283	−6.1	−0.93 (.000004)
***(2a) Tone-change (control) condition***	*No significant activation*				
***(2b) Tone-change (control) condition (with covariates*** * ***)***	*No significant activation*				

* Covariates included were IQ, Sound blending, and Auditory working memory scores.

### Genomic Procedures

Genomic DNA was extracted from buccal swab samples using QIAamp DNA mini-kit (Qiagen). *MCPH1* G37995C and *ASPM* A44871G genotypes were determined by polymerase chain reaction (PCR) amplification of DNA followed by direct sequencing of PCR products. Primers used for PCR amplification of *MCPH1* G37995C were 5′-TTTCAAAGGAAGAAATAAACTTGC-3′ and 5′-GAGGTGAATGGGAGCCATGT-3′; *ASPM* A44871G was amplified using primers 5′-AGGGCTGCAGTTCTCATTCAG-3′ and 5′-GCCCACTGAAGCTTTTGGTAG-3′. PCR conditions included denaturation at 94°C for 3 min followed by 45 cycles at 94°C for 30 sec, 55°C for 45 sec, and finally 72°C for 1 min. For each subject and for each gene, the number (load) of derived alleles was calculated. The G and C allele are derived alleles for *ASPM* A44871G and *MCPH1* G37995C, respectively. For both SNPs, we calculated the load of derived alleles for each gene in each subject. Therefore, the load of derived alleles for *ASPM* A44871G is 0, 1, and 2, for AA, AG, and GG alleles, respectively. The load of derived alleles for *MCPH1* G37995C is 0, 1, and 2 for GG, GC, and CC, respectively. For *ASPM*, we found 14, 12, and 6 subjects with the AA, AG, and GG genotype, respectively (Non-fMRI subjects: 7, 8, and 4 subjects with AA, AG, and GG genotype, respectively; fMRI subjects: 7, 4, and 2 subjects with AA, AG, and GG genotype, respectively). For *MCPH1*, there were 10 and 22 subjects with the GC and CC genotype, respectively (Non-fMRI subjects: 6 and 13 subjects with CC and GC genotype, respectively; fMRI subjects: 4 and 9 subjects with CC and GG genotype, respectively. There were no subjects with the GG genotype for *MCPH1* in either sample.). When both genes were examined together, there were 6, 12, 8, and 6 subjects with 1, 2, 3, and 4 combined derived alleles, respectively (Non-fMRI: 3, 7, 5, 4 subjects for 1, 2, 3, and 4 combined derived alleles, respectively; fMRI-subjects: 3, 5, 3, 2 subjects for 1, 2, 3, and 4 combined derived alleles, respectively). Both of our samples (fMRI and non-fMRI subjects) were in Hardy-Weinberg equilibrium. All statistical analyses in this report are based on two-tailed tests.

## Results

We estimated the correlation between Tone Perception performance and derived allele load for each gene. We used non-parametric tests as our primary analysis because the distributions were non-normal for both *ASPM* load [Kolmogorov-Smirnov Z  =  1.556, p  = .016] and *MCPH1* load [Kolmogorov-Smirnov Z  =  2.455, p <.001].

**Figure 2:**
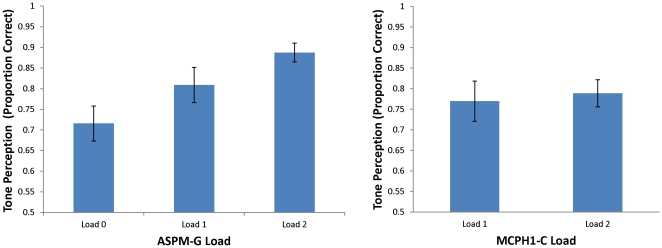
Relationship between Tone Perception and load of *ASPM-G* allele (left panel) and *MCPH1-C* allele (right panel). Significant genotype-phenotype correlation was found only in the *ASPM-G* allele.

We found a significant positive correlation between Tone Perception and the load of derived alleles for *ASPM* A44871G [Spearman’s Rho  =  0.433, p  =  0.013], even after age, SB, and AWM were partialed out [r (27)  =  0.448, p  =  0.015] (Figure 2). No significant correlations were found between the load of derived alleles for Tone Perception and *MCPH1* [Spearman’s Rho  =  0.051, p  =  0.781] or combined *MCPH1/ASPM* [Spearman’s Rho  =  0.343, p  =  0.055]. Furthermore, no significant correlations between other measures (age, and SB and AWM scores) and these two genes were found. When subjects who participated in the fMRI experiment were analyzed separately from those who did not participate in the fMRI experiment, there remained a significant correlation between the load of derived alleles for *ASPM* and Tone Perception [Non-fMRI subjects: Spearman’s Rho  =  0.695, p  =  0.001; fMRI subjects: Spearman’s Rho  =  0.557, p  =  0.048]. No such correlation between Tone Perception and the load of derived alleles for *MCPH1* was found in either sample [Non-fMRI subjects: Spearman’s Rho  =  0.124, p  =  0.613; fMRI subjects: Spearman’s Rho  =  0.0, p  =  1]. For combined *MCPH1/ASPM,* significant correlation was found only in the Non-fMRI subjects [0.579, p  = .009], but not in the fMRI subjects [0.387, p  = .192].

For the *ASPM* and Tone Perception correlation results, we used Fisher’s combined probability test [32] for calculating the probability of conducting a Type 1 error 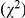 for two (i  =  2) consecutive samples (non-fMRI and MRI samples) using the formula 
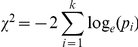
 where p equals the p-value obtained for each sample and found 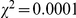.

**Figure 3:**
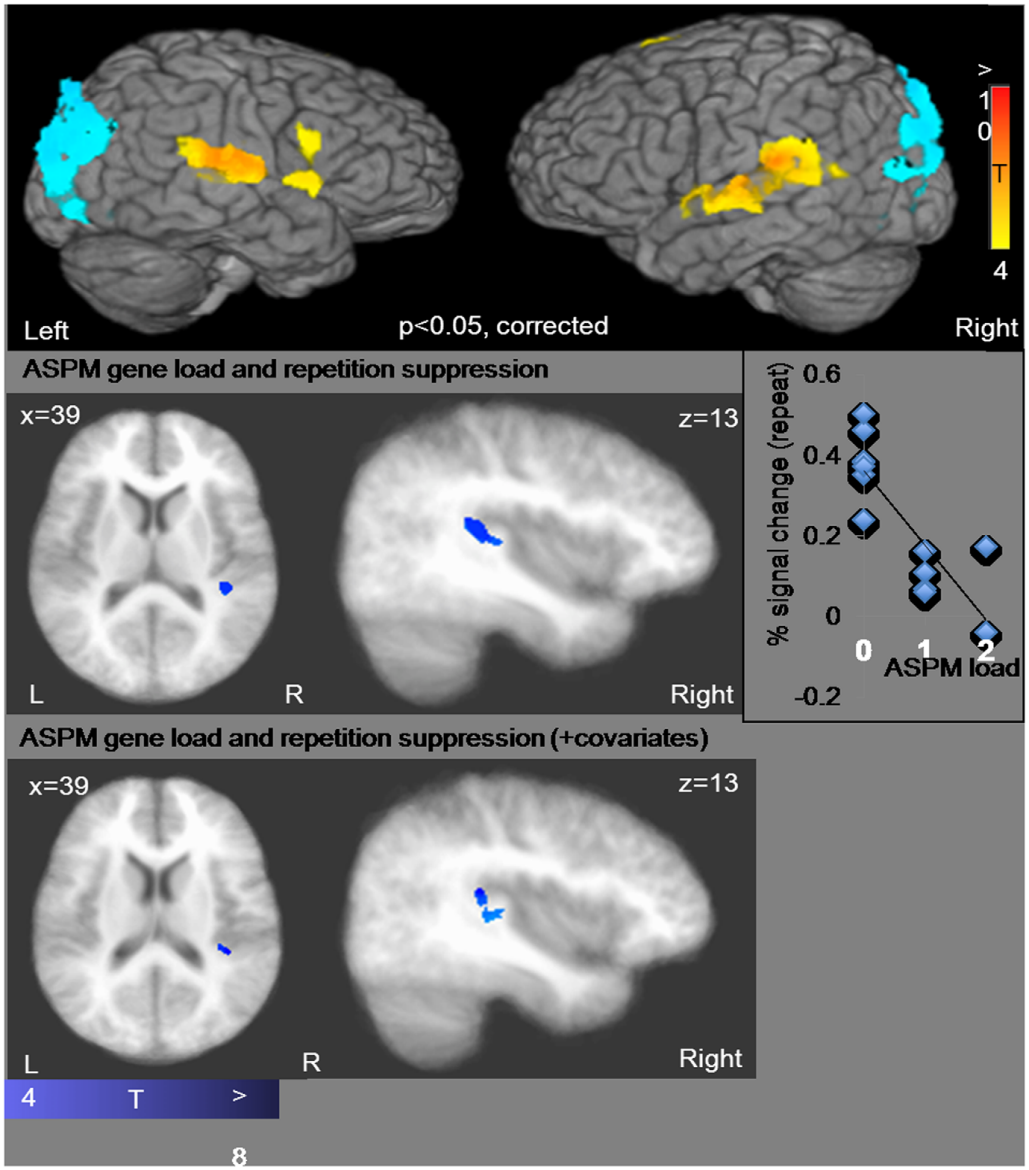
Relationship between load of *ASPM*-G allele and neural tone suppression in temporal cortex. Top panel shows bilateral temporal (and left inferior frontal) activation when participants listened to linguistic tones (irrespective of relationship with genotype). Middle panel shows results from multiple regression analysis (conducted on the repeat condition). A negative relationship between load of *ASPM*-G allele and extent of fMRI adaptation to tone was found in the right primary and association auditory regions. For illustrative purposes (to depict the direction of the relationship), we extracted estimates from the significant clusters. As seen in the scatter plot (middle right panel), individuals with higher allele load showed more suppression (lower BOLD activity) when tones were repeated, indicative of more efficient neural processing to tone information. This relationship in the right temporal was present even when various cognitive factors (IQ, sound blending, auditory working memory scores) were added as covariates (bottom panel). The right temporal cortex (especially the right Heschl’s gyrus) has been shown in previous studies to be important for tone processing [35], [37].

The association between the derived allele of *ASPM* and the neurophysiological basis of tone perception was found predominately in the right temporal cortex (Figure 3), where primary and association auditory cortical regions are located. Listening to tones, irrespective of genotype, significantly activated bilateral temporal regions (Figure 3). For the repeat condition, regression analyses revealed that the extent of BOLD activity in the right temporal cortex was *negatively* associated with the load of derived alleles for *ASPM*. In contrast, no region showed significant relationship between BOLD activity and the load of the derived alleles for *MCPH1* or *MCPH1/ASPM*. The relationship between *ASPM* load and repetition suppression in the right temporal regions was significant even after controlling for IQ, SB, and AWM (Table 1) in the multiple regression analysis. Participants who had a greater load of the derived allele showed *more* repetition suppression to tones in primary and association auditory cortical regions that are known to be sensitive to spectral processing. Regression analyses on the tone-change condition did not yield significant neural associations (Table 1). Since tone information is the only difference between tone-change and repeat condition (all other speech-related information is the same between the two conditions), we can conclude that the relationship between BOLD activity and *ASPM* derived allele load in the repeat condition is related to neural processing of tones.

## Discussion

Until now, the hypothesis that the derived alleles of *ASPM* A44871G and *MCPH1* G37995C contribute to human cognitive functions has lacked experimental support. We argue that the reason for these null findings is due to a general focus on broad measures of cognitive and brain functions such as IQ and brain size. These broad measures are likely amalgamations of multiple genetic and environmental factors, evidenced by the fact that all the known genetic information to date can only explain 1% of the variance in IQ [33], [34]. In the present study, we followed findings from a population study [18] linking these two genes to the perception of lexical tone, a more specific cognitive (linguistic) function, by experimentally examining the connection between these genes’ polymorphisms and tone perception in a behavioral and fMRI experiment. We found such a connection with *ASPM* A44871G, even after IQ, age, working memory, and phonemic awareness performances were controlled. Although the sample size is small for the fMRI experiment, it provides preliminary evidence suggesting that the behavioral connection seems to be associated with neurophysiological responses in primary and association regions of the auditory cortex, consistent with previous studies examining the neural basis of pitch perception [19], [35]–[38]. Previous studies have used fMRI adaptation methods to index functional properties of neuronal populations subserving auditory and linguistic function [28], [31]. Here we find a strong association between the extent of fMRI adaptation to lexical tone in the primary and secondary auditory regions, and the load of the derived allele of *ASPM.* Since no such relationship was found in the tone-change condition (tone information is varied), we can conclude that this relationship is specific to the encoding of tones. Furthermore, no such relationship was found with *MCPH1.*

Although the association between *ASPM*/*MCPH1* and more specific measures of language has been examined [7], their focus was on reading and spelling rather than speech sound perception. Similar to their study, we did not find an association with a measure associated with the mental manipulation of phonemes (Sound Blending), which is foundational to literacy skills [39], suggesting that these two genes are unrelated to phoneme manipulation per se. The fact that no connection was found with language deficits [7] (dyslexia and specific language impairment) further speaks to the hypothesis that these derived alleles contribute to normal variations of cognitive abilities and higher-level brain functions, unlike the effects of their deleterious mutations that cause primary microcephaly [1], [40], [41]. Most importantly, these alleles contribute to normal variations of specific linguistic abilities; in our case, we found evidence for tone perception to be at least one of these specific abilities.

Our study contributes to the current literature on the molecular genetics of human communication by not only considering its relationship with deficits [42]–[45], but also normal variations. All of our subjects scored within normal limits on all cognitive measures we assessed and had no reports of communication deficits. Furthermore, our study contributes to the understanding of the molecular genetics of pitch-related behaviors (e.g., music) and deficits (congenital amusia), which thus far have been found to have a genetic origin in twins [46], [47] and family-aggregation [48] studies.

It is worth pointing out that the present study was designed to examine two populations of subjects tested during two consecutive periods of time. We first conducted a behavioral study linking *ASPM/MCPH1* with lexical tone perception. After we found a significant correlation between *ASPM* and tone perception in this sample, we recruited a second group with the fMRI experiment added. While the sample size for the present study may appear small, it is consistent with human imaging genetic studies [49], [50]. Not only have we replicated our behavioral findings in two somewhat distinct populations, we have found converging evidence from both behavioral and neuroimaging experiments. As discussed in the Results section, the probability of conducting a Type I error for our behavioral results across our two consecutive samples was calculated based on Fisher’s method of combined probability test [32] and were found to be 0.0001. For the fMRI experiment, the p-values for the significant correlations we found between gene and auditory brain activation were very small (Table 1).

The lack of a significant association between *MCPH1* and our phenotypes could be due to our small sample size. It could also be related to the fact that we have a somewhat skewed distribution for the derived C allele. No subjects were homozygous for the ancestral G allele. Because the *ASPM/MCPH1* haplogroup depends on the distribution of the *MCPH1*group, a skewed *MCPH1* distribution could have also affected the results.

Dediu and Ladd [18] hypothesized that the presence of the derived allele of *ASPM* is associated with lexical tone and this association can either favor or disfavor tone. Other research groups hypothesize more specifically that the derived allele contributes a positive effect on human brain functions [8], [9]. Similar to Dediu and Ladd, our primary goal is to identify an association between *ASPM* and tone irrespective of the direction of the relationship. Interestingly, we found a positive relationship, whereas a negative relationship was found by Dediu and Ladd. The significant but opposite pattern of results between Dediu and Ladd and the present study could be attributed to differences in methodology. Dediu and Ladd [18] analyzed data collected from a population study that examined numerous populations of differing derived allele frequencies (e.g., 50% in French vs. 10.7% in Japanese), whereas we studied only one cultural group in order to minimize impacts of population stratification demonstrated in other experimental studies [51], [52]. Numerous studies have reported that the same allele could have differential phenotypic effects owing to factors such as gene-gene [53] and culture-gene interactions [54], [55] in different cultural groups. For example, American carriers of the GG/AG allele of the oxytocin receptor (OXTR) gene are reported to seek more emotional support than carriers of the AA allele, whereas Korean AA and GG/AG carriers did not differ [54]. Future studies should examine how these interactions may contribute to genotype-phenotype connections regarding *ASPM* and tone perception behaviors by examining different cultural groups.

In addition to potential contribution of gene-gene and culture-gene interaction effects, other factors related to inter- and intra-population comparisons might have contributed to differences in our results. Dediu and Ladd examined populations of subjects who acquire their native languages as children, whereas we examined individual differences in adults. Experience-driven effects started from a young age could affect genetic contribution to a phenotype that we could not observe in the present study. Furthermore, language differences at the population level are results of numerous historical and geographical factors, which could not be easily compared with effects of individual differences.

It is worth noting that although earlier research focusing on the SNPs that we examined in *ASPM* and *MCPH1* did not find a significant association between those SNPs and neural and cognitive phenotypes, some recent studies focusing on other SNPs of the same genes found significant results. Rimol et al. [2010] found sex-dependent associations between neuroanatomical measures and non-exonic SNPs of these two genes in an ethnically homogeneous Norwegian discovery sample and an ethnically heterogeneous North American replication sample. Similarly, Wang et al. [2008] found in Chinese males an association between cranial volume and a non-synonymous SNP of *MCPH1* other than the SNP that we examined. Future studies should examine the contribution of these SNPs and sex-dependency to tone perception.

An obvious limitation to the current study is the small sample size (n  =  32 for the main behavioral finding), although it is consistent with a number of previous studies connecting higher-order functions and genetics [46]–[47] Nevertheless, our current findings provide a starting point for examining a number of questions related to *ASPM.* For example, future research can examine whether *ASPM* and spoken language processing is restricted to lexical tones, to all speech sounds, to all speech sounds requiring simultaneous processing within a short temporal window, or whether they are also linked to other specific linguistic functions such as syntactic processing in a larger population of participants. Future studies can also examine other linguistic and speech-specific phenotypes.
